# The Association Between Psoriasis and Metabolic Syndrome in Children: A Narrative Review

**DOI:** 10.3390/metabo15060377

**Published:** 2025-06-06

**Authors:** Mateusz Matwiejuk, Hanna Myśliwiec, Agnieszka Mikłosz, Adrian Chabowski, Iwona Flisiak

**Affiliations:** 1 Department of Dermatology and Venereology, Medical University of Bialystok, 15-089 Białystok, Poland; hanna.mysliwiec@gmail.com (H.M.);; 2 Department of Physiology, Medical University of Bialystok, 15-089 Bialystok, Poland

**Keywords:** psoriasis, metabolic syndrome, children, diabetes mellitus, hypertension, dyslipidemia, obesity

## Abstract

Psoriasis is a common inflammatory skin disease with a complex pathogenesis consisting of genetic factors, immune dysfunction and environmental background. In adults, psoriasis is strongly associated with a higher risk of developing metabolic abnormalities; however, data in children are inconclusive. Metabolic syndrome (MetS) is a group of conditions that include central and abdominal obesity, hypertension, dyslipidemia and hyperglycemia. Potential pathogenic mechanisms linking psoriasis with metabolic syndrome include releasing large amounts of proinflammatory cytokines such as interleukins (IL-17, IL-23) and tumor necrosis factor alpha (TNF-α). These abnormalities promote excessive keratinocyte proliferation and impaired differentiation, which leads to typical psoriatic skin lesions. This paper aims to assess the potential link between psoriasis and each component of metabolic syndrome in children. It is speculated that the same proinflammatory cytokines produced by Th17 cells are also implicated in the development and progression of various metabolic disorders in patients with a severe course of the disease. Psoriatic patients are at higher risk for development metabolic diseases such as diabetes mellitus and cardiovascular disease.

## 1. Introduction

Psoriasis, one of the most common inflammatory skin diseases, affects ~2–3% of the general population and roughly 1% of children. It is a chronic disease that can occur at any age, but, importantly, children are also susceptible [1]. In the UK, approximately 0.55% of children aged 0–9 years and approximately 1.37% of children aged 10–19 years have psoriasis [2]. In children, a higher prevalence of psoriasis was described in girls (0.44%) compared to boys (0.35%) [3]. Henseler and Christophers proposed a classification system for psoriasis that distinguished two subtypes based on family history and certain genetic markers. The first is the familial subtype, with an early age of onset (<40 years), which is often associated with HLA-Cw6, DR7, B13 and B57; the second is the nonfamilial form, with a late age of onset form that is associated with HLA-Cw2 and B27 [4]. Psoriasis has several clinical manifestations, with the most common forms in children being plaque psoriasis (71%) and guttate psoriasis (26%) [5]. Children with plaque psoriasis typically present different characteristics compared to adults, like smaller plaques with finer and softer scale [6]. In turn, guttate psoriasis is characterized by small, widespread, erythematosus, scaly, tear-drop-shaped (guttate) plaques and papules, which appear abruptly [7].

Psoriasis is also a risk factor for the development of other serious and chronic diseases. Of particular interest is the association between psoriasis and metabolic syndrome (MetS). It was shown that hypertension, dyslipidemia, diabetes mellitus and obesity are more common in children and adolescents with psoriasis compared to their healthy peers. The prevalence of these conditions can be two-fold higher in pediatric psoriatic patients compared to healthy controls [8]. There is convincing evidence supporting an association between childhood psoriasis and an increased risk of developing MetS. The National Cholesterol Education Program Adult Treatment Panel III (NCEP ATP III) criteria are widely used to define metabolic syndrome. A person meets the criteria for metabolic syndrome if they have at least three of the following: a waist circumference of over 40 inches for men and 35 inches for women, a blood pressure of 130/85 mmHg or higher, a fasting triglyceride level of 150 mg/dL or higher, a fasting HDL cholesterol level of less than 40 mg/dL for men and less than 50 mg/dL for women, and a fasting blood sugar level of 100 mg/dL or higher [9]. In comparison to children, for adolescents 10–15 years of age, MetS is defined by the presence of at least one of the following: a waist circumference in the >90th percentile, systolic blood pressure (SBP) > 130 mmHg, diastolic blood pressure (DBP) > 85 mmHg, triglycerides > 150 mg/dL, HDL-C < 40 mg/dL. Importantly, for adolescents >15 years of age, the adult criteria for MetS diagnosis should be used. Interestingly, children <10 years should not be diagnosed with MetS, as has been argued by the lack of age-specific criteria for MetS components [10].

**Figure 1:**
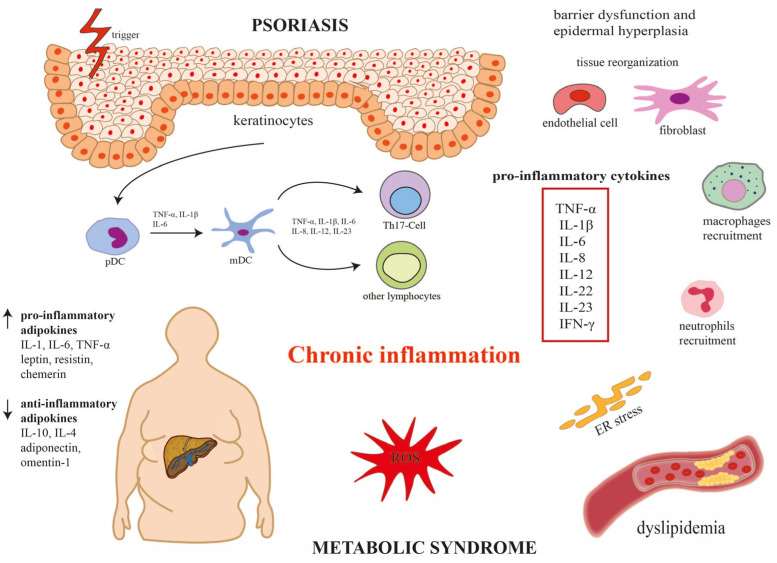
Potential mechanism underlying psoriasis and metabolic syndrome.

The hallmark of psoriasis is systemic inflammation. Recent advances in biological therapies have revealed that dysregulation of the IL-23/Th-17 immune signaling pathway plays a dominant role in the pathogenesis of psoriasis. Elevated levels of proinflammatory cytokines, such as interleukins (IL-17, IL-23), tumor necrosis factor alpha (TNF-α) disrupt keratinocyte differentiation and hyperproliferation. Chronic inflammation is also a well-known factor contributing to the development of metabolic disorders and similar immunological and inflammatory changes predispose to the development of psoriasis. For example, IL-17 promotes vascular inflammatory response, and in keratinocytes, it induces the transcription of several inflammatory cytokines (i.e., IL-1β, IL-6, IL-8 and TNF-α). Some of them, such as IL-8, act as chemoattractant for neutrophils, while the chemokine C-C-Motif Ligand (CCL)20 induces the recruitment of IL-17 producing cells, resulting in the acceleration of the inflammatory cascade. Furthermore, TNF-α, a paracrine mediator of cardiovascular diseases, plays an important role in the pathophysiology of inflammatory dermatoses. Anti-TNF-α therapies improve metabolic parameters and reduce cardiovascular complications in psoriatic patients [11]. In addition to increased release of proinflammatory cytokines, alterations in adipokines levels, oxidative stress, endoplasmic reticulum stress and dysbiosis of gut microbiota contribute to the development of MetS in psoriatic patients. The interaction of adipocytes with the immune system through various mediators, such as adipokines, may explain how obesity contributes to the development of psoriasis. Obese patients with psoriasis had low adiponectin and high leptin levels that were positively associated with the severity of psoriasis [12,13]. Figure 1 presents a potential mechanism underlying psoriasis and metabolic syndrome. Although patients with psoriasis may have an increased incidence of metabolic syndrome, this association is still under investigation, and there are many studies with conflicting results. This narrative review aims to critically evaluate the potential association between childhood psoriasis and each component of MetS, examining proposed pathophysiological pathways and clinical implications.

Dysregulation of the IL-23/Th-17 immune signaling pathway plays a dominant role in the pathogenesis of psoriasis and increases the incidence of metabolic syndrome. In the skin, affected keratinocytes activate dendritic cells (DCs), and under the influence of tumor necrosis factor α (TNF-α), interleukins (IL-1β, IL-6, IL-8, IL-12, IL-23) stimulate T helper 17 cell (Th17) differentiation. Activated Th17 cells secrete a variety of proinflammatory cytokines (i.e., TNF-α, IL-1β, IL-6, IL-8, IL-12, IL-22, IL-23, INF-γ), leading to barrier dysfunction and epidermal hyperplasia and the development of chronic inflammation. Changes in adipokine levels (i.e., increase in proinflammatory adipokines and decrease in anti-inflammatory adipokines), oxidative stress, and endoplasmic reticulum stress contribute to the development of metabolic syndrome in patients with psoriasis. IL-1β, interleukin 1β; IL-6, interleukin 6; IL-8, interleukin 8; IL-12, interleukin 12; IL-22, interleukin 22; IL-23, interleukin 23; INF-γ, interferon gamma; mDC, myeloid dendritic cell; pDC, plasmacytoid dendritic cell; Th17, T helper 17 cells.

## 2. Materials and Methods

A medical literature search of PubMed (1992–present), conducted in the winter of 2025, was performed using appropriate terms without date limitations. The main object of the research was to identify the association between psoriasis and components of metabolic syndrome. Medical subject headline terms included “psoriasis and hypertension”, “psoriasis and cardiovascular diseases”, “psoriasis and high blood pressure”, “psoriasis and obesity”, “psoriasis and dyslipidaemia”, “psoriasis and hypercholesterolemia”, “psoriasis and hyperglycemia”, “psoriasis and diabetes mellitus”. Non-English publications, papers with low clinical significance, papers written in a language other than English, and duplicated publications were excluded from the analysis. Originally, human and animal studies were included in this narrative review. The results of the search strings were combined together, and duplicates were removed. Afterwards, the titles and abstracts of the studies searched were independently screened by two reviewers (M.M. and H.M.) in order to identify relevant articles that addressed the review subject. Disagreements between reviewers were resolved by a fourth reviewer (A.C.). Finally, the selected eligible articles were fully reviewed.

## 3. Discussion

### 3.1. Metabolic Syndrome in Children with Psoriasis

Caroppo et al. [14] showed that children with psoriasis more often have metabolic syndrome (30%) and insulin resistance (27%) compared to healthy children. Children with MetS had significantly higher: body weight, body mass index (BMI), waist circumference (WC), waist-to-height ratio (WHtR), prevalence of overweight/obesity and prevalence of central obesity in comparison to children without MetS. All of the above-mentioned anthropometric differences were statistically significant (*p* < 0.004, *p* < 0.001, *p* < 0.001, *p* < 0.001, *p* = 0.002, *p* < 0.001, respectively). In turn, duration, the severity, and familial history of psoriasis did not differ significantly between children with and without MetS (*p* > 0.1) [14]. See Table 1.

Similarly, Goldminz et al. [15] observed that children with psoriasis had a higher incidence of metabolic syndrome compared to healthy children. However, no significant correlation was found between MetS and Psoriasis Area and Severity Index (PASI) or Body Surface Area (BSA). This indicates that the severity of psoriasis, as measured by these indices, does not directly correlate with the presence of MetS. Moreover, no significant difference was found in BMI percentiles between both groups. In turn, regression analysis showed significant correlations between MetS criteria and waist circumference percentile (r = 0.70), BMI percentile (r = 0.66), HDL-C (high-density lipoprotein cholesterol) (r = 0.66), triglycerides (r = 0.50), SBP (r = 0.40) and DBP (r = 0.39) [15]. See Table 1.

Tollefson et al. [16] reported that a large group of almost 30,000 children with psoriasis had a higher rate of MetS, including large waist, hyperlipidemia, hypertriglyceridemia, hypertension (HTN), and type 2 diabetes mellitus (T2DM) [16]. See Table 1.

Torres et al. [17] reported a higher prevalence of MetS in children with psoriasis (25%) compared with control children (3.7%). Patients with psoriasis had a significantly higher mean BMI percentile (*p* = 0.018) and were overweight or obese (*p* = 0.03). The age- and sex-adjusted odds ratio for excess adiposity was 4.4 (95% CI 1.2–15.6), indicating a significantly increased risk of overweight/obesity in children with psoriasis. Interestingly, there were no significant differences in hypertension, hypercholesterolemia, hypertriglyceridemia, diabetes, or insulin resistance between the two groups. There was also no statistically significant difference in the overall lipid profile. However, ox-LDL (oxidized low-density lipoprotein) and apoB (apolipoprotein B) levels were higher in the psoriasis group, although these differences did not reach statistical significance (*p* = 0.113 and *p* = 0.247, respectively). Despite the lack of overall significance, a trend toward an increased prevalence of metabolic syndrome was observed in children with psoriasis. Two specific components of metabolic syndrome were significantly higher in the psoriasis group: waist circumference (*p* = 0.002) and systolic/diastolic blood pressure (SBP/DBP) > 90th percentile (*p* = 0.032) [17]. See Table 1.

Al Mutairi et al. [18] showed that MetS was more common in the group that did not receive TNF inhibitors (50.42%) compared to the group that received them (41.52%). This difference was statistically significant, with an OR of 1.76 (95% CI: 1.19–2.41; *p* = 0.005). Firstly, increased waist circumference was observed in 56.77% of children treated with TNF inhibitors. Secondly, triglyceridemia was less common in children treated with TNF inhibitors. Thirdly, fasting blood glucose and serum triglycerides were significantly lower in the group receiving TNF inhibitors. These results suggest that treatment of psoriasis with TNF inhibitors may have a protective effect against the development of some components of metabolic syndrome in children with psoriasis [18]. See Table 1.

Au et al. [19] observed a higher prevalence of MetS in children with psoriasis compared to controls [19]. See Table 1.

Lakshmi et al. [20] confirmed that there is a potential association between childhood psoriasis and MetS. Furthermore, in children with psoriasis and metabolic syndrome, the mean duration of psoriasis was longer (4.75 years) compared with children without metabolic syndrome (1.44 years) [20]. See Table 1.

Lee et al. [21] noticed a higher prevalence of MetS in Australian children with psoriasis (8%) compared to the control group (0%). The authors observed a trend towards a higher prevalence of MetS in children with moderate to severe psoriasis (20%) compared to those with mild psoriasis (3%). This suggests a potential association between psoriasis severity and the risk of MetS [21]. See Table 1.

Mhusakuncha et al. [22] suggested that metabolic syndrome may be more common in older children (over 12 years of age) with psoriasis compared with younger children (under 12 years of age). Children over 12 years of age with psoriasis had a higher prevalence of metabolic syndrome (27 out of 70 cases, or 0.38) compared with children under 12 years of age (25 out of 107 cases, or 0.23) [22]. See Table 1.

Kelati et al. [23] shed light on several factors that may increase the risk of MetS in children with psoriasis. They found that the prevalence of metabolic syndrome in the studied pediatric population was 3.7%. Children with more extensive or severe psoriasis (pustular or erythroderma types) were found to be at higher risk of MetS. Nail involvement, facial involvement, psoriasis refractory to topical therapy, and poor quality of life were also associated with an increased risk of MetS in children with psoriasis [23]. See Table 1.

Mahe et al. [24] found no association between the age of psoriasis onset and the prevalence of cardiovascular disease and MetS in later adulthood [24]. See Table 1.

Ranugha et al. [25] found that mixed facial psoriasis was the most common type of facial psoriasis. This likely means that psoriasis affects areas such as the forehead, around the ears, and potentially even the nose and mouth. Moreover, there is a strong association between mixed facial psoriasis and a higher score on the Psoriasis Area Severity Index (PASI). Interestingly, the study did not find a significant relationship between facial involvement (including mixed facial type) and MetS [2]. See Table 1.

Pinter et al. [26] found that screening for psoriasis in children appears to psoriatic arthritis (PsA) over MetS, although these data suggest a higher risk of MetS in this population. MetS may not be as widely recognized as a risk factor in children with psoriasis as in adults [26]. See Table 1.

### 3.2. Hypertension in Children with Psoriasis

Tollefson et al. [16] found that children with psoriasis were at increased risk of developing hypertension (HR, 1.64; 95% CI, 1.40–1.93) than children without psoriasis. The only comorbidity for which a significant interaction between psoriasis and obesity was observed was HTN (*p* = 0.03). While obesity increased the risk of HTN in all children, this effect was less pronounced in children with psoriasis. Children with psoriasis already had an elevated baseline risk of hypertension. In children without psoriasis, obesity increased the risk of HTN 7.27-fold. In children with psoriasis, obesity increased the risk 4.15-fold (HRs of 6.81 for obese and 1.64 for non-obese children). This meant that obese children with psoriasis had an HR of 6.81, and non-obese children with psoriasis had an HR of 1.64 [16]. See Table 2.

Caroppo et al. [27] found that children with psoriasis had a higher prevalence of both high SBP and DBP compared to children without psoriasis [27]. See Table 2.

Jensen et al. [28] revealed that children and adolescents with psoriasis had higher SBP, but not DBP compared to controls. Peripheral arterial tonometry (PAT) measurements in children and adolescents with mild to moderate psoriasis were similar to those in healthy controls. This also supports the observation that mild to moderate psoriasis may not significantly impair endothelial function, as indicated by PAT [28]. See Table 2.

Kwa et al. [29] found that Black and/or Hispanic children with psoriasis had the highest risk of developing HTN in comparison to healthy children [29]. See Table 2.

Augustin et al. [1] reinforced the concept that juvenile psoriasis is linked to a higher risk of developing HTN compared to children without psoriasis [1]. See Table 2.

Kimball et al. [30] noticed that HTN was significantly more common in the psoriasis cohort than in the psoriasis-free control subjects over the entire observation period [30]. See Table 2

### 3.3. Dyslipidemia in Children with Psoriasis

Panjiyar et al. [31] revealed that patients with moderate to severe psoriasis had lower high-density lipoprotein cholesterol (HDL-C) levels compared to those with milder psoriasis or the control group [31]. See Table 3.

Tom et al. [32] found that children with psoriasis had lower levels of HDL-C particles compared to healthy children [32]. See Table 3.

Moudgil et al. [33] noticed that children with psoriasis were significantly more likely to have low HDL-C compared to controls [33]. See Table 3.

Toruniowa et al. [34] conducted a study on lipid profiles in children with psoriasis. Reduced HDL-C levels and increased TG levels have been observed in children with psoriasis [34]. See Table 3.

Ferretti et al. [35] reported that psoriatic children had a significant increase in the composition of low-density lipoprotein cholesterol (LDL-C) and HDL particles compared to healthy children [35]. See Table 3.

In contrast to the above-mentioned findings, Ferretti et al. [36] found that children with psoriasis had higher total cholesterol levels and HDL-C cholesterol in comparison to the control group [36]. See Table 3.

Interestingly, the study by Al Mutairi et al. [37] showed that tofacitinib treatment for children with psoriasis led to changes in their lipid profile, specifically an increase in LDL-C and HDL-C levels, which was maintained throughout the 36-week study period [37]. See Table 3.

Koebnick et al. [38] showed that overweight or obese adolescents with psoriasis had higher levels of total cholesterol, triglycerides, and LDL-C in comparison to patients without psoriasis [38]. See Table 3.

The study by Simonetti et al. [39] found that psoriatic prepubertal children had higher plasma of total cholesterol and HDL-C compared to controls [39]. See Table 3.

Shreberk-Hassidim et al. [40] showed that adolescents with psoriasis had significantly higher TG levels compared to those without psoriasis [40]. See Table 3.

Murzina et al. [41] revealed that children with psoriasis, regardless of the BMI- (normal or elevated), had lipid profiles that were in the reference range **and** were similar to the lipid profiles of the control group [41]. See Table 3.

### 3.4. Hyperglycemia in Children with Psoriasis

Burgmann et al. [42] showed that only 2 out of 369 adolescents with type 1 diabetes mellitus (T1DM) suffered from psoriasis [42]. See Table 4.

A study by Michalak et al. [43] showed that patients with psoriasis had a milder onset of type 1 diabetes (T1DM). First, they had higher serum C-peptide levels at diagnosis, indicating better endogenous insulin secretion. Interestingly, six months after T1DM diagnosis, patients with psoriasis had slightly lower HbA1c levels compared to those without psoriasis. HbA1c is a marker of long-term blood sugar control, reflecting average glucose levels over the previous 3 months [43]. A case study by Ojiami et al. [44] showed a potential association between psoriasis, specific symptoms (headache, granulomatous panuveitis), diabetic ketoacidosis and the development of type 1 diabetes. Interestingly, the 11-year-old child in the case study suffered from Vogt–Koyanagi–Harada (VKH) disease [44]. See Table 4.

Kluger et al. [45] identified an earlier association between VKH and psoriasis [45]. See Table 4.

Di Costanzo et al. [46] noted that psoriasis was found in 4.7% (9 out of 191) of the children with T1DM. All patients had plaque psoriasis with a mean PASI score of 2.4, indicating moderate psoriasis [46]. See Table 4.

### 3.5. Obesity in Children with Psoriasis

Hunjan et al. [47] showed that obese children are at higher risk of developing psoriasis. Moreover, the severity of psoriasis was positively correlated with the severity of obesity. The odds ratio was 2.58, meaning that children with psoriasis had a 2.58-fold increased likelihood of being obese than children without psoriasis. However, for children who were not obese at the time of psoriasis diagnosis, the odds of developing obesity after diagnosis were similar to those for children without psoriasis. Additionally, the study found that children diagnosed with psoriasis had a considerably higher risk of developing arthritis than children without psoriasis, HR = 8.17 [47]. See Table 5.

Similarly, Ergun et al. [48] also found a significant association between psoriasis and being overweight or obese. Psoriatic patients had a higher prevalence of obesity (28%) compared to the control group (19%). Ergun’s research suggests that being overweight/obese does not directly worsen psoriasis severity or interfere with the effectiveness of topical medications. Furthermore, the study found no significant association between overweight/obesity and disease progression. However, disease duration was a significant factor—that is, the longer a person had psoriasis, the more likely they were to progress from a localized to a generalized psoriasis [48]. See Table 5.

Guidolin et al. [49] reported that children with psoriasis had a 3.16-fold increased likelihood of being obese (based on BMI) and a 5.84-fold increased likelihood of having central obesity (based on waist-to-height ratio (WtHR)) compared to children without psoriasis [49]. See Table 5.

Lee et al. [21] found that children with psoriasis had a significantly higher prevalence of elevated WtHR than the control group, indicating a greater tendency for central accumulation of adipose tissue in these children [21]. See Table 5.

Mahe et al. [24] showed an association between childhood psoriasis and a higher prevalence of overweight with abdominal obesity (58% of the studied cases), which was significantly associated with the severity of psoriasis and psoriatic arthritis. Children with childhood-onset psoriasis (COP) have a significantly lower BMI compared with adults with adult-onset psoriasis (AOP). COP is also characterized by a significantly lower waist circumference. The prevalence of overweight and obesity is also significantly lower in patients with COP [24] (see Table 5). See Table 5.

Choon et al. [50] observed a trend towards higher odds of obesity (defined as BMI less than or equal to the 85th percentile) in children with psoriasis compared to non-inflammatory controls (OR = 2.35, 95% CI: 0.99–5.56). However, there was no significant difference in the risk of obesity between children with psoriasis and those with eczema (OR = 1.14, 95% CI: 0.5–2.62) [50]. See Table 5.

In contrast to the above-mentioned studies, Manos et al. [51] found no statistically significant difference in the prevalence of obesity among children with psoriatic arthritis, children from the psoriasis group and healthy control children [51]. See Table 5.

## 4. Conclusions

In this narrative review, we present the current understanding of the association between psoriasis and metabolic syndrome in children. Psoriasis is often associated with increased blood pressure, increased fasting glucose, excess waistline fat, and abnormal cholesterol and triglyceride levels. This is likely due to the fact that psoriasis and metabolic syndrome share many common inflammatory and cytokine-mediated mechanisms. In general, children with psoriasis have a higher incidence of metabolic syndrome, and patients with more severe psoriasis have a higher risk of developing metabolic syndrome than patients with milder psoriasis. However, differences in the study population, such as age, ethnicity, lifestyle, and medication use, may mask a potential association between psoriasis and metabolic syndrome. Therefore, a large population-based study is needed to determine the exact association between psoriasis and metabolic syndrome in children, including the environmental, genetic, and immunological factors leading to their co-occurrence.

## Figures and Tables

**Table 1:** Summary of the studies on metabolic syndrome in children with psoriasis.

Author	Year	Mean Age	Population	Key Observation
		Metabolic Syndrome in Children with Psoriasis		
Caroppo et al. [14]	2021	n1—7.71 ± 2.4 years n2—7.8 ± 2.4 years	n1—42 children without MetS n2—18 children with Mets	Children with psoriasis had significantly higher body weight, body mass index (BMI), waist circumference (WC), waist-to-height ratio (WHtR), prevalence of overweight/obesity, and prevalence of central obesity.
Goldminz et al. [15]	2013	n—13.5 ± 2.7 years n1—13.5 ± 2.7 years	n—20 healthy patients n1—20 patients with psoriasis	Children with psoriasis had a higher incidence of metabolic syndrome compared to healthy children
Tollefson et al. [16]	2018	n—12.07 years n1—12.07 years	n—29 957 patients without psoriasis n1—29 957 patients with psoriasis	A large group of nearly 30,000 children with psoriasis had higher rates of MetS.
Torres et al. [17]	2014	n—10.4 ± 2.87 years n1—10.40 ± 3.15 years	n—27 control patients n1—20 patients with psoriasis	Patients with psoriasis had a significantly higher mean BMI percentile (*p* = 0.018) and were overweight or obese.
Al Mutairi et al. [18]	2018	n1—12.7 ± 3.8 years n2—13.1 ± 2.4 years	n1—236 patients with psoriasis treated with anti-TNF inhibitors n2—236 patients with psoriasis treated without anti-TNF inhibitor’s treatment	Anti-TNF treatment shows promise in potentially reducing this association between psoriasis and MetS.
Au et al. [19]	2012	n/a	n1—1536 control patients n2—20 patients with psoriasis	Higher prevalence of MetS in children with psoriasis compared to controls.
Lakshmi et al. [20]	2023	n/a	n1—32 patients with psoriasis	There is a potential association between childhood psoriasis and MetS.
Lee et al. [21]	2016	n1—9.3 years n2—8.3 years	n1—73 controls n2—135 patients with psoriasis	A higher prevalence of MetS in Australian children with psoriasis compared to the control group.
Mhusakunchai et al. [22]	2021	n1—10.5 years	n1—177 patients with psoriasis	MetS may be more common in older children (over 12 years of age) with psoriasis compared with younger children (under 12 years of age).
Kelati et al. [23]	2017	n1—11.5 years n2—11.2 years n3—14.2 years	n1—64 psoriasis with metabolic comorbidities n2—20 children with non-metabolic comorbidities n3—76 children with no comorbidity	Children with more extensive or severe psoriasis (pustular or erythroderma types) were found to be at higher risk of MetS.
Mahe et al. [24]	2013	n/a	n—2201 psoriatic patients	No association has been found between the age of psoriasis onset and the prevalence of cardiovascular disease and MetS in later adulthood.
Ranugha et al. [25]	2021	n/a	n1—62 psoriatic patients without facial involvement n2—188 psoriatic patients with facial involvement	No significant link between facial involvement (including mixed facial type) and MetS.
Pinter et al. [26]	2020	n/a	n1—95 patients with psoriasis	MetS may not be as widely recognized as a risk factor in children with psoriasis.

Abbreviations: n—total group, n1 = control group, n2—study group, CVD—cardiovascular disease, HTN—hypertension, T2DM—diabetes mellitus type 2, PASI—Psoriasis Area Severity Index, PsA—psoriatic arthritis.

**Table 2:** Summary of the studies on hypertension in children with psoriasis.

Author	Year	Mean Age	Population	Key Observation
		Hypertension in Children with Psoriasis		
Tollefson et al. [16]	2018	n—12.07 years n1—12.07 years	n—29 957 patients without psoriasis n1—29 957 patients with psoriasis	Children with psoriasis were at an increased risk of developing hypertension.
Caroppo et al. [27]	2019	n—10.60 ± 2.98 years n1—10.60 ± 2.98 years	n—52 healthy patients n1—52 psoriatic patients	Children with psoriasis had a higher prevalence of both SBP and DBP compared to children without psoriasis.
Jensen et al. [28]	2014	n1—14.3 ± 2.1 years n2—14.5 ± 2.3 years	n1—30 healthy patients n2—30 psoriatic patients	Children and adolescents with psoriasis had higher SBP, but not DBP, compared to controls.
Kwa et al. [29]	2017	n/a	n—1606 children with psoriasis	Black and/or Hispanic children suffering from psoriasis have the highest odds of developing HTN in comparison to healthy ones.
Augustin et al. [1]	2009	n/a	n1—1 310 090 patients without psoriasis n2—33 981 patients without psoriasis	Juvenile psoriasis is linked to a higher risk of developing HTN compared to children without psoriasis.
Kimball et al. [30]	2012	n1—11.4 ± 4.1 years n2—11.4 ± 4.1 years	n1—37 020 patients without psoriasis n2—7404 patients with psoriasis	HTN were significantly more common in the psoriasis cohort than in the psoriasis-free control subjects.

Abbreviations: SBP—systolic blood pressure, DBP—diastolic blood pressure.

**Table 3:** Summary of the studies on dyslipidemia in children with psoriasis.

Author	Year	Mean Age	Population	Key Observation
		Dyslipidemia in Children with Psoriasis		
Panjiyar et al. [31]	2023	n1—10.77 ± 3.90 years n2—11.54 ± 3.94 years	n1—52 healthy patients n2—52 psoriatic patients	Patients with moderate to severe psoriasis had lower HDL-C levels.
Tom et al. [32]	2016	n1—13.0 ± 4.3 years n2—13.0 ± 4.3 years	n1—44 control patients n2—44 psoriatic patients	Children suffering from psoriasis had lower levels of HDL-C.
Moudgil et al. [33]	2021	n1—10.6 ± 2.91 n1—1.37 ± 2.7	n1—50 healthy patients n2—104 psoriatic patients	Children with psoriasis had low HDL-C.
Toruniowa et al. [34]	1994	n/a	n/a	Reduced HDL-C levels and increased TG levels have been observed in children with psoriasis.
Ferretti et al. [35]	1994	n/a	n1—14 healthhy children n2—15 psoriatic children	Psoriatic children had significant increases in the composition of their LDL-C and HDL particles.
Ferretti et al. [36]	1993	n1—9 ± 2.3 years n2—9.4 ± 2.2 years	n1—16 healthhy children n2—15 psoriatic children	Children with psoriasis had higher total cholesterol levels and a specific increase in HDL-C cholesterol.
AlMutairi et al. [37]	2020	n—12.3 years	n—47 patients with psoriasis	Tofacitinib treatment in psoriatic children led to changes in their lipid profile, specifically an increase in both LDL-C and HDL-C levels.
Koebnick et al. [38]	2011	n/a	n1—132 831 patients without psoriasis n2—439 patients with psoriasis	Overweight or obese adolescents with psoriasis had higher levels of total cholesterol, triglycerides, and LDL-C.
Simonetti et al. [39]	1992	n1—6 ± 4.1 (males) and 8.3 ± 2.7 (females) n2—9.19 ± 2.59 (males) and 9.43 ± 2.10 (females)	n1—27 healthy patients n2—30 psoriatic patients	Psoriatic prepubertal children had higher plasma of total cholesterol and HDL-C levels.
Shreberk-Hassidim et al. [40]	2019	n/a	n1—884 653 healthy patients n2—3112 psoriatic patients	Adolescents with psoriasis had significantly higher TG levels.
Murzina et al. [41]	2020	n1, n2—12.11 ± 0.44 years	n1—77 psoriatic patients with BMI in normal limits n2—31 psoriatic patients with BMI abve normal limit	Children with psoriasis, regardless of BMI (normal or elevated), had lipid profiles that fell within the reference range.

Abbreviations: n/a—not applicable, HDL-C—high-density lipoprotein cholesterol, LDL-C—low-density lipoprotein cholesterol, BMI—body mass index.

**Table 4:** Summary of the studies on impaired glucose levels in children with psoriasis.

Author	Year	Median Age	Population	Key Observation
		Impaired Glucose Levels in Children with Psoriasis		
Burgmann et al. [42]	2020	n—12.3 ± 4.4 years	n—369 patients with T1D	Overall, 2 out of 369 adolescents with T1DM suffered from psoriasis.
Michalak et al. [43]	2017	n1—11.28 years n2—13.17 years	n1—129 healthy patients n2—14 psoriatic patients	Patients with psoriasis had a milder onset of type 1 diabetes mellitus.
Ojiami et al. [44]	2012	n/a	n—11-year-old child with psoriasis	There is a potential association between psoriasis, specific symptoms (headache, granulomatous panuveitis), diabetic ketoacidosis and the development of type 1 diabetes.
Kluger et al. [45]	2008	n/a	n—49-year-old Italian woman	Presumably, there is a relationship between VKH and psoriasis.
Di Costanzo et al. [46]	2017	n—12.3 years	n—191 consecutive T1D outpatients	Psoriasis was found in 4.7% (9 out of 191) of the children with T1DM.

Abbreviations: T1D—type 1 diabetes, VKH—Vogt–Koyanagi–Harada syndrome.

**Table 5:** Summary of the studies on obesity in children with psoriasis.

Author	Year	Median Age	Population	Key Observation
		Obesity in Children with Psoriasis		
Hunjan et al. [47]	2018	n/a	n1—1100 healthy patients n2—550 psoriatic patients	Obese children are at higher risk of developing psoriasis. The severity of psoriasis was positively correlated with the severity of obesity.
Ergun et al. [48]	2016	n1—10.2 years n2—11.6 years	n1—151 healthy patients n2—289 psoriatic patients	There is a significant association between psoriasis and being overweight or obese.
Guidolin et al. [49]	2018	n1—9.94 ± 3.48 years n2—9.94 ± 3.48 years	n1—107 healthy patients n2—107 psoriatic patients	Children with psoriasis had a 3.16-fold increased likelihood of being obese and a 5.84-fold increased likelihood of having central obesity.
Lee et al. [21]	2016	n1—9.3 years n2—8.9 years	n1—73 controls n2—135 patients with psoriasis	Children with psoriasis had a significantly higher prevalence of elevated WtHR than the control group.
Mahe et al. [24]	2013	n/a	n—2201 psoriatic patients	There is an association between childhood psoriasis and a higher prevalence of overweight with abdominal obesity.
Choon et al. [50]	2016	n2, n3—11.0 years n4—13.5 years	n2—56 non-inflammatory skin diseases n3– 59 patients with atopic eczema n4—92 patients with psoriasis	There is a trend towards higher odds of excess adiposity in children with psoriasis.
Manos et al. [51]	2017	n1, n3—n/a n2—8.8 ± 5.2	n1—909 healthy patients n2—48 patients with PsA n3—231 patients with psoriasis	No differences were observed in the incidence of obesity among children with psoriatic arthritis, children from the psoriasis group and healthy control children.

Abbreviations: WtHR—waist-to-height ratio.

## Data Availability

No new data were created or analyzed in this study.
